# Celiac Disease in Moroccan Children: Diagnostic Characteristics and Determinants of Diagnosis Delay

**DOI:** 10.7759/cureus.50800

**Published:** 2023-12-19

**Authors:** Assia Mouslih, Karima El Rhazi, Nassiba Bahra, Mounia Lakhdar Idrissi, Moustapha Hida

**Affiliations:** 1 Laboratory of Epidemiology and Health Research, Faculty of Medicine and Pharmacy, Sidi Mohammed Ben Abdellah University, Fez, MAR; 2 Department of Pediatrics, Faculty of Medicine and Pharmacy/ Epidemiology and Health Science Research Laboratory, Hassan II University Hospital, Fez, MAR

**Keywords:** diagnosis delay, diagnosis age, symptom onset age, children, celiac disease

## Abstract

Advances in the field of celiac disease have led to a better understanding of the disease, but it remains underdiagnosed and poses a daily challenge to clinicians to make a timely diagnosis. This study aims to analyze and describe diagnosis characteristics, diagnosis delay, and the factors influencing this delay in Moroccan children.

Our study included 324 children diagnosed during the study period from January 01, 2010, to December 30, 2019, at the Department of Pediatrics, Hassan II University Hospital in Fez, Morocco. Data were collected using a collection grid and then analyzed using SPSS 26 software (IBM Corp., Armonk, NY).

The results showed a female predominance (n=197, 60.8%), with a diagnosis age of 73.8±46.8 months. The mean age onset of symptoms was 51.3±41.2 months, and the diagnosis delay was 22.2±22.6 months, with only 32.7% (n=106) diagnosed less than 12 months after symptom onset.

The most common consultation reason was diarrhea (n=149, 46%) and growth delay (n=105, 32.4%) and 50.5% (n=98) of parents consulted a pediatrician first. The three clinical, serologic, and histologic criteria made it possible to agree on the diagnosis, with the clinical profile dominated by the digestive form at 84.9% (n=279), serologic with the presence of IgA transglutaminase antibodies (95.7%; n=310), and histologic with villous atrophy at 91.7% (n=297). Unfortunately, 14.8% (n=48) of the children were diagnosed with a celiac crisis.

The multivariate logistic regression analysis showed that as symptoms onset age increased, so did the risk of late diagnosis (OR=0.96, 95% CI: 0.94 to 0.97, p<0.001). Age of diagnosis was also associated with delayed diagnosis (OR=19.68, 95% CI: 8.77 to 44.15, p<0.001).

The combination of these variables and the diagnosis delay argues in favor of adopting a diagnosis strategy that includes raising awareness among healthcare professionals of the need to identify typical and atypical cases early in order to reduce the adverse effects of late diagnosis and the complications that can result.

This methodology for improving diagnoses may also unearth previously unknown aspects of celiac disease in Moroccan children.

## Introduction

Celiac disease (CD) is an autoimmune, systemic, and chronic enteropathy resulting from exposure of a genetically predisposed individual to a food antigen represented by gluten proteins found in wheat, barley, and rye [1-3]. Its pathogenesis is caused by the interplay of genetic, immunological, and environmental factors, which induce an immune response in the intestinal mucosa through the action of HLA molecules [2].

CD is evolving into a significant issue for global public health [1]. Epidemiological knowledge about the illness has drastically changed in recent years [4]. It is transforming from a very rare disease to one of the most common health challenges facing children [5]. It is now considered the most common inflammatory bowel disease; its frequency in the population has increased from 0.02% in 1975 [6] to a higher prevalence of 1% in the general population [7, 8]. However, it is still underestimated and mistakenly diagnosed as in most cases among children, CD is silent, paucisymptomatic, or atypical [5].

CD epidemiology has iceberg characteristics [9]. Depending on the region, there are significant differences in the prevalence of CD worldwide [10]. The disease’s prevalence in the general population varies significantly between countries, ranging from 0.7 to 2% [11, 12]. CD is not limited to developed countries [13]. Recent epidemiological studies in developing countries have revealed prevalence rates comparable to those in Europe [14]. In the Americas, Australia, South-West Asia, and North Africa, recent epidemiological studies have found prevalence rates similar to European statistics [10]. Every year, 42,000 children die from complications related to diarrhea, with undiagnosed CD accounting for approximately 4% of all diarrheal deaths [15].

The clinical presentation of children is quite varied and heterogeneous in terms of symptoms and severity [11, 12]. The symptoms are frequently divided into gastrointestinal and non-gastrointestinal categories [16]. The presence of enteropathy evidenced by persistent diarrhea with nutritional and stature consequences [13,14] is a classic definition of CD based on this triad [17], but other clinical presentations are also possible [4]. One of the factors contributing to misdiagnosis is the variety of clinical presentations [12].

For over 25 years [12,18], CD presentation has evolved [19] due to the advent of new serological markers that allow for the detection of CD with high sensitivity and specificity [12,18]. However, the identification of anti-transglutaminase (tTG) antibodies, which are the most widely used serologic markers and which revealed frustrating clinical manifestations [4], has enabled significant advances in CD diagnosis [20,21]. Despite these advances, the disease remains severely underdiagnosed [17]. Even in developed countries, on average, five cases go undiagnosed for each one diagnosed [13], while in developing countries, diagnosis is still based on symptomatic forms [22]. Undiagnosed CD in childhood has serious consequences in adulthood [20,23], leading to short stature and low female fertility [24]. This should encourage the optimization of diagnostic conditions in children by generalizing autoantibody testing, which is currently the first step of the disease's diagnosis [20]. In a context like ours [22], CD is a diagnosis problem [17,25], and its impact is widely underestimated [22]. In Morocco, epidemiologic studies are lacking; the diagnosis of typical and atypical forms of CD and their frequency are still to be determined [12,13], and the prevalence of CD is unknown [26].

In our country, there are few studies on CD in children. A study presented in 2013 on a series of children in Casablanca presented a comparison of clinical aspects over time after the occurrence of serologic tests [27]. Another study in Marrakech in 2016 presented the experience of the Pediatrics Department of the University Hospital of Marrakech in the care of celiac children [25]. Finally, in 2021, a study was carried out on the quality of life among children with CD [28]. To our knowledge, none of these studies has investigated delayed diagnosis and its determinants.

The present study, conducted in the pediatric department of the Centre Hospitalier Universitaire (CHU) Hassan II in Fez, has the ambition to fill this gap. It aims to describe the clinical, serological, and histological characteristics and the determinants of the delay in the diagnosis of CD in children.

## Materials and methods

Study design

This descriptive-analytical study was conducted among children diagnosed in the gastrointestinal unit located in the pediatric department of the CHU Hassan II in Fez, Morocco, between January 1, 2010, and December 30, 2019. Three clinical, serological, and histological criteria were used to identify CD in the 324 child subjects of this study.

Data collection 

Data was collected over three months using a data collection sheet form. Sociodemographic characteristics such as gender and origin were recorded. Regarding pre-diagnosis history, the presence of CD in the family as well as the symptom onset age in months was recorded, the latter was defined as the time between diversification and symptoms onset (the time between the age at which gluten was introduced into the diet and the onset of the symptoms).

The reason for consultation, which represents the first symptoms observed by the family that led to the child's consultation in a health facility, was also collected. The diagnoses were confirmed based on clinical, biological, and anatomopathological data, including the age of the patient.

Clinical symptoms were classified into digestive (diarrhea, abdominal pain, bloating, constipation, and trouble in transit), and extra-digestive symptoms such as growth delay. Weight and height were systematically measured and plotted on WHO growth charts. The weight and height evolution was expressed in standard deviation (SD), where a child's growth was considered normal if the variables (weight and height) evolved in the same corridor between +2 and -2. Other extra-digestive symptoms were also identified: delayed puberty, irritability, chronic fatigue, and anemia. Celiac crisis was identified as a severe, acute form of the disease with symptoms such as acute diarrhea, edema syndrome, dehydration, and malnutrition, usually requiring intensive hospitalization.

Comorbidity data was also collected regarding type I diabetes, hypothyroidism, lactose intolerance, epilepsy, GH deficiency, and dermatitis herpetiformis.

Serological analysis tests used to verify antibody positivity were anti-transglutaminase IgA/IgG, anti-endomysium IgA/IgG, and antigliadin IgA/IgG. To confirm the positive serological diagnosis, the type of serologic tests collected from the files were those that have shown positivity.

Anatomopathologic data was used to confirm the diagnosis. The Marsh classification standard was used to determine intestinal lesions, which was as follows: Marsh 0 (no atrophy), Marsh I (lymphocytic infiltration), Marsh II (signs of lymphocytosis and cryptic hyperplasia), Marsh III (A: partial atrophy, B: subtotal atrophy, C: total atrophy), Marsh IV (total atrophy with hypoplasia (flat mucosa). A Marsh score of II, III, or IV is compatible with CD. However, in our study, even stage I with a clinical picture was considered as CD.

The time to CD diagnosis was calculated by subtracting the diagnosis age from the symptom onset age. The studied population median of the time to CD diagnosis was 12 months, and this variable was divided into two groups. The first group called “early diagnosis” involved a diagnosis time of <12 months and the second group called “delayed diagnosis” had cases that required ≥12 months to confirm their diagnoses.

Ethical guidelines

Data collection was done in compliance with the Declaration of Helsinki's ethical guidelines. The protocol was submitted to the Ethics Committee of the Faculty of Medicine and Pharmacy. The committee's approval was granted under number 12/22.

Statistical analysis

The collected data were imported into Excel and analyzed using SPSS 26 (IBM Corp., Armonk, NY). Quantitative variables were outlined using descriptive statistics, in the form of a mean, standard deviation, or median. Numbers and percentages were used to represent qualitative factors.

The association between relevant factors and delayed diagnosis was investigated using the chi-square and Fisher's test. A value of p<0.05 was considered statistically significant, and the confidence interval was set at 95%.

Variables found to be significant by univariate analysis were entered into a multivariate logistic regression model to determine factors associated with diagnosis delay, with adjustments for confounders.

## Results

In total, 324 children diagnosed with CD were included in this study. They were predominantly female (n=197, 60.8%), with a sex ratio of 1.5 F/M, but no difference in the time to CD diagnosis was found (p=0.69). Most of them 83.3% (n=270) were from the Fez-Meknes region, with 54.7% (n=148) living in Fez, and no association between residence and time to diagnosis was observed (p=0.5).

The mean diagnosis age was 73.8±46.8 months, with a peak diagnosis at age 64.3 months (5 years and 3 months). Before the age of five years, 49.5% of cases were diagnosed. The mean time to diagnosis was 22.2±22.6 months (range: 1-120). Of these, 67.2% (n=147) were diagnosed late. Diagnosis age in children was statistically associated with delayed diagnosis (p<0.01).

The mean symptoms onset age was 51.3±41.2 (range: 3-192) with a mode of 12 months. The mean time between diversification and symptom onset was 35.4±34.4 months (range: 0-162) (Table 1). This diversification caused an immediate onset of symptoms in 8% (n = 13), whereas there was an association between time to CD diagnosis and symptom onset age (p = 0.04) and time between diversification and symptom onset (p = 0.02).

**Table 1:** Descriptive of variables in children with celiac disease Plus or minus values are means ± standard deviation (SD)

Variables (in months)	Mean±DS	Range	95% CI
Diagnosis age	73.79±46.86	6-204	68.67 to 78.98
Symptoms onset age	51.30±41.24	3-192	46.25 to 56.35
Time to diagnosis	22.23±22.66	1-120	19.46 to 25.00
Diversification age	6.44±2.16	3-18	6.12 to 6.76
Time between diversification and symptoms onset	35.42±34.43	0-162	30.10 to 40.75

The presence of CD in the family was noted in 4.6% (n=15), where 46.7% (n=7) were siblings, but the presence of the disease did not influence the diagnosis delay (p=0.6).

According to the data collected, the most common reason for consultation was diarrhea (n=149, 46%), followed by growth delay (n=105, 32.4%). Also, children who consulted for digestive symptoms had a greater chance of being diagnosed within one year of symptom onset than children who manifested extra-digestive symptoms. However, children with growth delay have a statistical association with delayed diagnosis (p<0.01).

After noticing the first symptoms, 50.5% (n=98) of parents first consulted a pediatrician, while 22.2% (n=43) went to the emergency room. However, the first visit was not associated with a diagnosis delay (p=0.7). 

Diagnosis characteristics

When diagnosed, 58.7% (n = 190) of patients had two or three symptoms at the same time, while 14.5% (n=47) had only one symptom. The clinical presentation was divided into digestive symptoms (n=279, 84.9%) and extra-digestive symptoms (n=249, 76.9%). Digestive manifestations were dominated by diarrhea (n=185, 57.1%) followed by abdominal distention (n=124, 38.3%) and vomiting (n=64, 19.8%). Concerning extra-gastrointestinal symptoms, our study population presented a wide range of symptoms, dominated by growth delay (n=150, 46.3%), followed by weight loss (n=36, 11.1%), and anemia (n=53, 16.4%). However, asthenia (n=21, 6.5%), pallor (n=6, 1.9%), and muscular hypotonia (n=4, 1.2%) were also present in low proportions.

In our population, most children (n=273, 84.3%) were underweight, while 81.8% (n=265) were stunted, with the standard deviation of weight and height having a median and mode of -2.

The analysis showed that digestive symptom type was not statistically associated with delayed diagnosis (p=0.37), whereas extra-digestive symptoms were not (p=0.02). Certain symptoms of each type were statistically associated with delayed diagnosis (Table 2).

**Table 2:** Comparison of time to diagnosis with clinic-pathologic features of pediatric celiac disease *p-value<0.05 is considered statically significant ** Plus or minus values are means ± standard deviation (SD)

Variables	Time to Diagnosis		p
	Early diagnosis < 12 months	Delayed diagnosis >=12 months	
	N(%)/ mean ± SD	N(%)/ mean ± SD	
Range age (in months)			<0.01*
6-24	39 (46)	15 (8.6)	
24-60	27 (31.7)	51 (29.3)	
60-144	13 (15.3)	96 (25.2)	
144-204	6 (7)	12 (6.9)	
Digestive symptoms	77 (33.7)	151 (66.3)	0.37
Diarrhea	51 (31.9)	109 (68.1)	0.62
Vomiting	28 (50)	28 (50)	0.002*
Abdominal pain	11 (22)	39 (78)	0.07
Bloating	42 (36.2)	74 (63.8)	0.06
Extra-digestive symptoms	60 (30.5)	137 (69.5)	0.14
Growth delay	27 (24.5)	83 (75.5)	0.01*
Anemia	16 (35.6)	29 (64.4)	0.6
Consultation reason			<0.01*
Digestive	63 (40.4)	93 (59.6)	
Extra-digestive	21 (21.4)	77 (78.6)	
Comorbidity	8 (26.7)	22 (73.3)	0.4
Symptoms onset age^**^	44.00±43.76	26.98±33.87	0.04*
Time between diversification and symptoms onset^**^	26.98±33.87	39.96±34.02	0.02*

In this study, 14.8% (n=48) of patients were diagnosed with celiac crisis and were hospitalized for a mean duration of 10±7 days (range: 2-32 days). Watery diarrhea (n=15, 31.3%), dehydration (n=11, 22.9%), undernutrition (n=10, 20.8%), edematous syndromes (n=6, 12.5%), and fever (n=5, 10.4%) were the most common reasons for hospitalization. Celiac crisis (p=0.24) did not have a significant impact on time to CD diagnosis.

The prevalence of comorbidity in diagnosed celiac children was 10.8% (n=35), with type 1 diabetes (n=26, 74.3%) being the most prevalent, followed by hypothyroidism (n=4, 11.4%). It should be highlighted that comorbidity was diagnosed in 60% (n=21) of children prior to the discovery of CD. However, there was no correlation between comorbidity and diagnosis delay in our research (p=0.4).

Serological Tests

The first-line serological tests were IgA and IgG tTG in 95.7% (n=310) and 52.2% (n=169) of children, AGA IgA and IgG in 6.8% (n=22) and 5.9% (n=19), and EMA IgA and IgG in 7.7% (n=25) and 3.4% (n=11) respectively. At the same time, two types of serology were performed in 50.9% (n=165) of children.

Anatomopathological Results

All serologically positive children benefited from gastro-duodenal fibroscopy. Pathological examination showed total villous atrophy in 85.5% (n=278) of children, of whom 75% (n=243) were stage III.

Multivariate analysis

Univariate analysis showed that the diagnosis delay was closely linked to the child's diagnosis age, the reason for consultation, the presence of extra-digestive symptoms (growth retardation), symptoms onset age, and time between diversification and symptoms onset.

Variables where univariate analysis yielded a p-value >0.2 were entered into a multivariate logistic regression model.

The multivariate analysis eliminated confounders and showed a significant difference between symptom onset age and diagnosis age in diagnosis delay (Table 3).

**Table 3:** Multivariate logistic regression analysis for diagnosis delay associated with symptoms onset age and diagnosis age Adjusted for symptoms onset age, diagnosis age, time between diversification and symptoms onset, extra-digestive symptoms, and consultation reason.

Significant variables	OR	95% CI	p-value
Symptoms onset age	0.96	0.94 to 0.97	<0.001
Diagnosis age	19.68	8.77 to 44.16	<0.001

## Discussion

The aim of this study was to describe the clinical, serologic, and histologic characteristics of a sample of Moroccan children diagnosed with CD. It also analyzed the diagnosis delay and the factors contributing to it. The main finding was a delay in diagnosis in 67.2% of cases. Multivariate analysis eliminated confounding factors and retained symptom onset age and age group at diagnosis as the main associated variables.

In our series, the effect of symptom onset age and diagnosis age on diagnosis delay was due to the effect of age at presentation on the diversity of symptoms [21]. This clinical variability and heterogeneity leads to misdiagnosis and delayed diagnosis [17,28-30]. Our findings are also explained by the myth that CD is still perceived as a pediatric disease [15], with a classic presentation that often manifests in the first years of life, often in the months following the introduction of gluten [19,29]. In fact, it is a systemic disease with atypical [31], and non-classical symptoms that is under-recognized by physicians [32].

At present, the cause of this variability in the clinical expression of CD is unknown [17], as is the cause of the onset of the symptoms at different times after gluten ingestion [33], given that some develop CD within months of gluten ingestion [29], whereas others may consume gluten for many years before the disease becomes apparent [17].

Univariate analysis also revealed an association between the number of symptoms and delay: the more heterogeneous the child's symptoms, the more delayed the diagnosis. Around 51.5% of our population had between two and four symptoms. Our result is in agreement with a study by D'amico et al. on children in the USA, who reported that almost half of the sample presented with three symptoms [34]. The wide variety of symptoms varies considerably over time from one child to another and even within the same child [17]. This diversity results in a broad clinical picture and exposes the child to a late or even missed diagnosis [35].

In our population, the mean diagnosis age was 6.1 years, which is between the age of the Marrakech series (5.8 years) [24], the Tunisian series (5.9 years) [36], the age in Mediterranean countries (5.9 years) [37], and between the mean diagnosis age of Turkish children (6.4 years) [38]. Nevertheless, our diagnosis age is high compared to that reported by Boudraa et al. (3.6 years) [5]. This shows a general trend towards late-onset CD in children (between five and seven years) [35].

More than half of our series were diagnosed before the age of five years. Similarly, 51.8% of the cases in the study by Hariz et al. were diagnosed within the first five years of life [36]. It is rare for CD to first manifest in adolescence [39], therefore only 7.3% of the children in our series were diagnosed in adolescence. However, Garnier et al. claim that the peak incidence occurs between one and two years of age [29].

Underdiagnosis is common worldwide [14,40,41]; it represents a challenge to the healthcare system [42,43] as diagnosis requires a high index of suspicion by clinicians [31]. Thus, the diagnosis of CD is often delayed [44] and late [29,45].

In children, the average delay varies from a few weeks to a few months [46]. In our study, the average delay was approximately two years, with a range of up to 10 years. Several studies have reported an average delay longer than ours, ranging from 3.5 to 14 years [41,45,47-53]. Our delay remains short compared to other studies, as diagnosis in our context is limited to classical forms of the disease [24].

However, 64% of patients in our study sought help from a pediatrician as their first port of call, but there was no association between diagnosis delay and the level of health care sought by the patient when symptoms first appeared [54].

The disease is clearly underdiagnosed [55,56]. The majority of cases go undiagnosed [49,57]. Even in developed countries [57], for every diagnosed case of CD, an average of four to seven cases remain undiagnosed [17,58,59]. In general, a significant proportion of CD cases remain undiagnosed, with rates ranging from 70-90% [10,42,60].

Late diagnosis can significantly reduce life quality [54,61,62], leave a patient suffering from a myriad of symptoms to develop serious complications while awaiting diagnosis [45,61], lead to refractory CD [63], and even increase the risk of lymphoma [64].

However, univariate analysis has shown an association between the type of reason for consultation and delayed diagnosis, which may be influenced by two elements: one element is related to the physician [50], where the knowledge of health professionals is unsatisfactory [34]; clinical identification could be a challenge for physicians [65] with a heterogeneous clinical picture having only minimal symptoms and even misleading symptoms [21,29,30,66,67]. The second element relates to the patient, who accepts a reduced state of well-being [54] and finds his condition tolerable, accepting it as a normal, chronic, and vaguely pathological state [68].

Although several studies report that celiac crisis is a rare presentation and that its occurrence is due to late diagnosis as a complication of celiac disease [69-72], our study showed that 14.8% were diagnosed with celiac crisis. This high proportion can be explained by late diagnosis.

Unexpectedly, growth retardation, one of the most characteristic and frequent symptoms of CD in children [73], increases the risk of late diagnosis. The 58.6% growth retardation found in our study is higher than the rate reported by Nardecchia et al., which ranges from 10-40% of cases [74] and may represent a significant burden for pediatric patients with implications for lifelong growth potential [39,75]. This association is due to the perception by clinicians that isolated growth retardation is not typical of CD.

Despite a female predominance [76,77], mainly explained by the high rate of autoimmune diseases in women in general [68], this predominance does not influence the delay in diagnosis [78-80], as demonstrated in our study.

The literature shows that celiac patients have a higher risk of developing an autoimmune disease [81], and the association between CD and autoimmune disease [28,82] is present in almost one-third of cases [28]. Szajewska et al. estimated the prevalence of pathologies potentially associated with CD in children to be 20.7% [83]. In our series, 10.8% had an associated disease. Knowledge of these associations allows earlier diagnosis [28,84]. Celiac patients are 5 to 10 times more likely to have type 1 diabetes than the general population [85]. In our population, the main associated pathology is T1DM (74.3%), and no association between long delay and the presence of type 1 diabetes has been demonstrated [85].

Serologic and histologic tests are used to confirm the diagnosis of CD [20]. In recent decades, a wide range of highly sensitive and specific serologic tests has become available [86], representing a major advance in the history of the disease [87], and the detection of circulating tTG has shown a higher frequency of disease than previously estimated [88].

Because of the greater accuracy, high sensitivity, and specificity of the IgA tTG test in the pediatric population, serology is usually initiated with this test [15]. In our series, serodiagnosis is also more specifically based on IgA tTG antibodies (97%), followed by IgG tTG antibodies (50.2%).

However, in our series, the anatomopathologic study confirmed the diagnosis in our population and showed that 92.6% had villous atrophy. In the Iranian series, biopsy showed that 90.4% of the studied population had villous atrophy of varying degrees [89], and histologic examination also showed atrophy in 92% of cases [90]. In contrast, a study from the United Arab Emirates showed histologic positivity in only 57.7% of cases [91].

At present, knowledge about the extent and factors involved in delayed diagnosis is still scarce and insufficiently clarified [49,50,54]. However, early diagnosis reduces mortality and the prevalence of disorders associated with CD [14], where mortality is almost four times higher in the undiagnosed population [92].

The key to preventing CD is currently unknown [15]. Primary prevention consists of avoiding the development of the disease [93]. Our results argue that primary prevention is not possible by acting on the age of symptom onset, as symptom onset is not an option [17]. Even the promotion of breastfeeding does not guarantee a sustained protective effect against the development of CD [75,76], whereas the timing of gluten introduction has a protective effect in at-risk children [94,95], especially if it is introduced while breastfeeding continues [37,96]. However, Lionetti et al. report that neither late introduction of gluten nor breastfeeding alters the risk of CD in at-risk infants [94].

Secondary prevention aims at early detection of the disease, thus increasing the possibility of interventions to halt disease progression and the onset of symptoms [93]. Despite existing guidelines, in practice, physicians face many difficulties in referring patients to appropriate tests for the diagnosis of CD [45] as CD is often misunderstood by physicians [97].

The diagnosis delay in our series remains unacceptable given the vulnerability of our population. Despite numerous studies, questions regarding delayed diagnosis remain unanswered [78]. In our context, possible explanations for the delay in diagnosis are (1) The erroneous belief that CD is rare and pediatric [91,98,99]; (2) lack of awareness of the variability of clinical presentation [37,75,85,91]; (3) difficulty of access to care in developing countries [45,57,100,101]; (4) lack of screening in at-risk individuals [58,75,85], and the nutritional and epidemiological transition that is currently undergoing in Morocco [102,103].

Limitations

The major limitation of the study is that it is based on the files of diagnosed children, which means that some data are missing. Another limitation is that the study is cross-sectional and monocentric, which limits the exploration of factors that may further explain the delay in diagnosis.

## Conclusions

In conclusion, despite the fact that diagnostic conditions have improved and that diagnosis in our context is dominated by classical forms, our study showed a delay in diagnosis in 67.3% of cases.

However, the current challenge is to establish a diagnostic strategy that will reduce the delay in diagnosis and reveal the other hidden faces of CD. This strategy must be based on the wider availability of serologic tests and the awareness among healthcare professionals of the clinical heterogeneity and age variability of CD onset.
